# Supplementary material for: Impaired Fear Extinction Recall in Serotonin Transporter Knockout Rats Is Transiently Alleviated during Adolescence
Source: Brain Sci. 2019 May 22;9(5):118. doi: 10.3390/brainsci9050118 (PMC6562656; doi:10.3390/brainsci9050118)
Supplement: Supplementary file 1 [file brainsci-09-00118-s001.pdf]

## Supplementary Figures

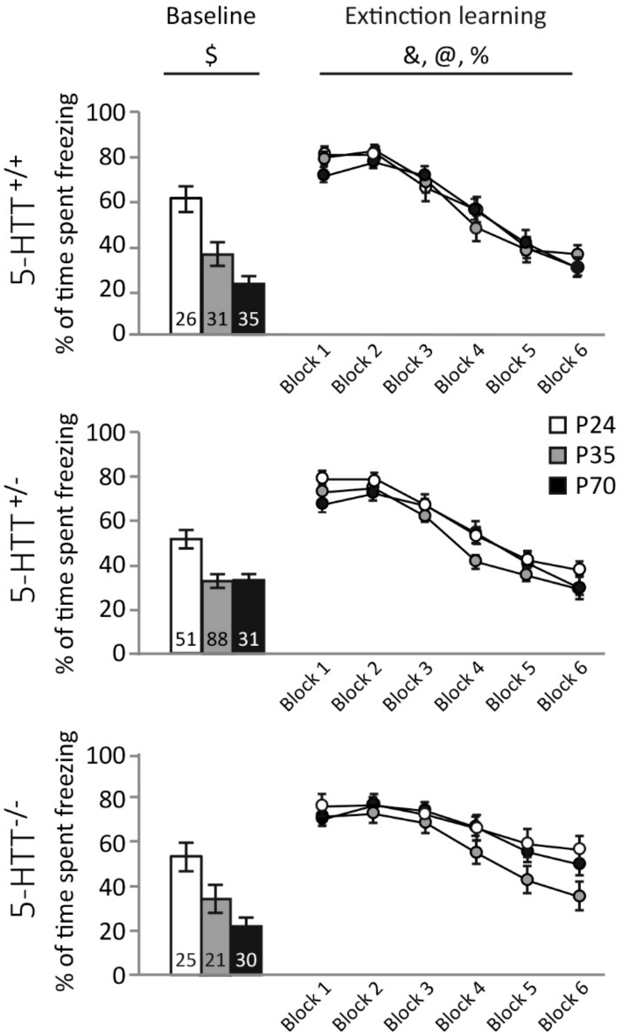


**Figure S1.** Fear conditioning behavioral data across extinction learning and the two extinction recall sessions, sorted on genotype. Data are expressed as mean % of time spent freezing during stimulus presentations ± standard error of the mean. $ indicates a significant effect of age ($p < 0.05$); ¥ indicates a significant age x genotype interaction ($p < 0.05$); & indicates a significant effect of extinction block ($p < 0.05$); @ indicates a significant age x block interaction ($p < 0.05$); % indicates a significant genotype x block interaction ($p < 0.05$).

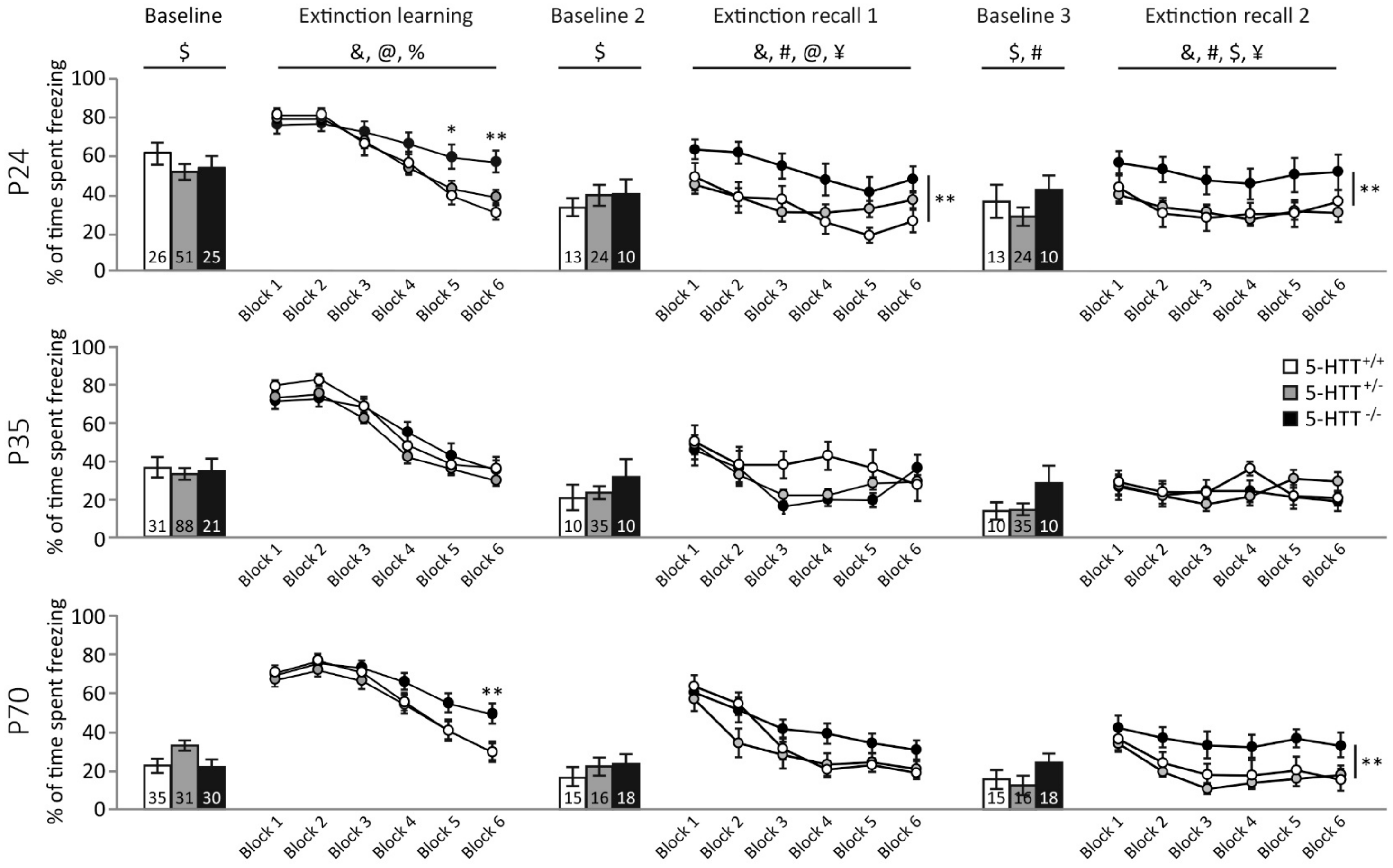


**Figure S2**. Fear conditioning behavioral data across extinction learning and the two extinction recall sessions, including baseline. Freezing during the 2-minute stimulus free baseline period preceding extinction learning decreased across age in all genotypes (see main text). Baseline freezing observed prior to the first extinction recall session also revealed a strong effect of age ($F_{(2,144)} = 6.597$, $p = 0.002$), with preadolescent animals showing highest freezing levels ($p = 0.006$ compared to adolescents; $p = 0.001$ compared to adults), whereas adolescent animals did not differ from adults ($p = 1.000$). Baseline freezing during the second fear extinction recall session also revealed a strong effect of age ($F_{(2,142)} = 9.063$, $p < 0.001$), with preadolescent animals showing highest freezing levels ($p < 0.001$ compared to adolescents; $p = 0.002$ compared to adults), whereas adolescent animals did not differ from adults ($p = 1.000$). Freezing rates were also modulated by genotype ($F_{(2,142)} = 3.970$, $p = 0.021$), with 5-HTT$^{-/-}$ rats displaying higher freezing than the other groups ($F_{(1,107)} = 8.281$, $p = 0.005$, compared to 5-HTT$^{+/-}$; $F_{(1,107)} = 2.983$, $p = 0.089$, compared to 5HTT$^{+/+}$), whereas 5-HTT$^{+/-}$ and 5HTT$^{+/+}$ rats did not significantly differ from each other in freezing behavior ($F < 1$). Data are expressed as mean % of time spent freezing during stimulus presentations ± standard error of the mean. # indicates a significant effect of genotype ($p < 0.05$); $ indicates a significant effect of age ($p < 0.05$); ¥ indicates a significant age x genotype interaction ($p < 0.05$); ** indicates a significant *post hoc* difference between 5-HTT$^{-/-}$ vs. 5-HTT$^{+/-}$ and/or 5-HTT$^{+/+}$ rats ($p < 0.01$); & indicates a significant effect of extinction block ($p < 0.05$); @ indicates a significant age x block interaction ($p < 0.05$); % indicates a significant genotype x block interaction ($p < 0.05$).

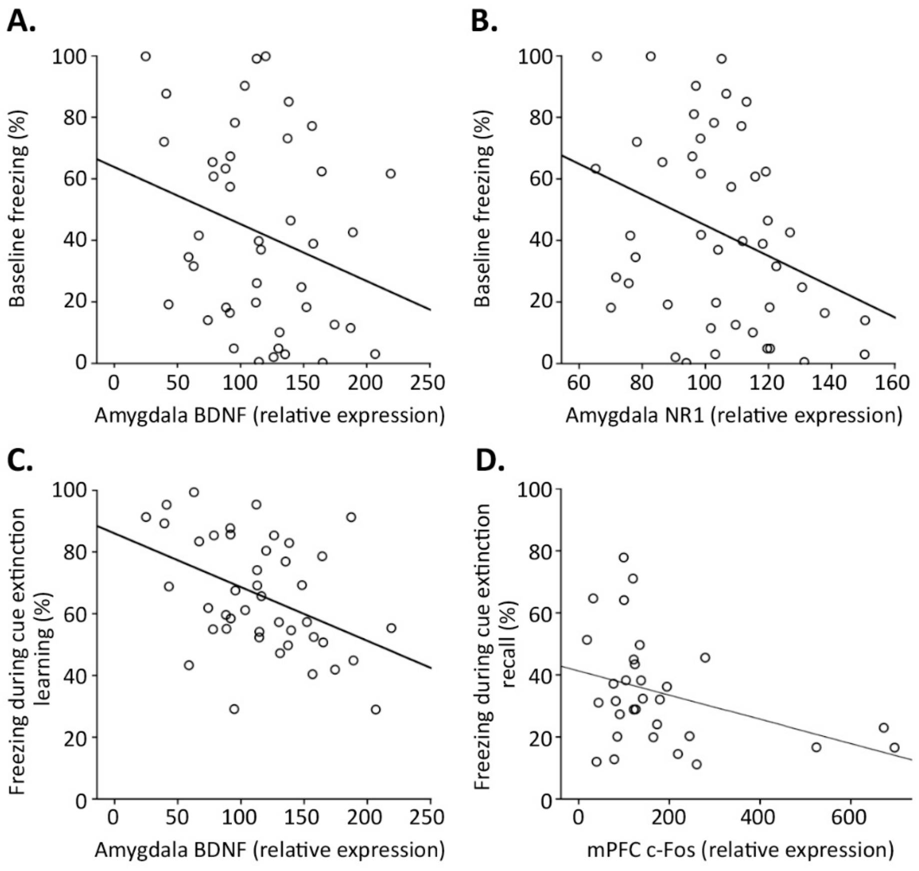


**Figure S3.** Correlational plots showing associations between brain gene expression levels and behavioral freezing. Baseline freezing as observed prior to fear extinction learning correlated significantly with amygdala BDNF (**A**) and NR1 (**B**) expression levels, whereas amygdala BDNF also correlated with freezing rates during cue presentation during this session (**C**). Freezing as observed during the second fear extinction recall session correlated with c-Fos expression in the medial prefrontal cortex (mPFC) (**D**). Gene expression levels are depicted as percentage of the respective preadolescent 5-HTT$^{+/+}$ group.
